# Infectious Disease Modeling and Epidemic Response Measures Analysis Considering Asymptomatic Infection

**DOI:** 10.1109/ACCESS.2020.3016681

**Published:** 2020-08-14

**Authors:** Xingguang Chen

**Affiliations:** 1 School of BusinessJianghan University 74777 Wuhan 430056 China; 2 Manufacturing Industry Development Research Center on Wuhan City CircleJianghan University 74777 Wuhan 430056 China; 3 Institute of Intelligent Decision-Making, Jianghan University 74777 Wuhan 430056 China

**Keywords:** Epidemics, asymptomatic infectious, modeling, evolution, response measures

## Abstract

Classical SIR dynamic model and its derivative improved model may not accurately describe the epidemic situation similar to COVID-19 with characteristics of relative long incubation period and a large number of asymptomatic infections. Based on the existing epidemic compartment model, a novel compartment dynamic model considering actual transmission path of the symptomatic and asymptomatic infected is presented. Theoretical analysis and numerical simulation are employed to conduct prediction of development of the epidemic. According to different epidemic response measures, i.e., mitigation measures, suppression measures, medical treatment, evolutionary trend of epidemic situation under the initial population distribution structure are discussed. Results show that the control effects of different response measures on the number of deaths depend on the timing of the implementation of the measures. For mitigation response measures, the timing of the implementation of the measures has no obvious effect on the final epidemic, while for suppression response measures, the effect of suppression response measures in the early stage of the epidemic is significantly better than that in the middle and late stage of the epidemic development. Furthermore, no matter which stage the epidemic is in, the improvement of medical treatment level will play an important role in effectively reducing mortality. This study provides useful enlightenment and decision-making reference for policy makers to choose appropriate epidemic prevention and response measures in practice.

## I. Introduction

Recently, the new coronavirus (SARS-CoV-2) has spread all over the world. How to prevent and control the epidemic effectively has become a global hotspot. Existing theoretical research on infectious disease modeling can be generally divided into three categories. The method used in the first category is mainly based on the method of random process, which usually uses the branching process of random process to explore the spread and evolution of infection population [1]–[5]. The second kind of method is mainly differential equation method, which is usually extended based on the compartmental model [6]–[14]. The third category mainly employs statistical physics methods, which usually consider disease -behavior interactions on complex networks [15]–[20]. For example, Ref. [1] used spreading processes to model the process of disease transmission, and investigated the effect of basic reproductive number R0 on the probability of disease emergence. On the basis of [3], Ref. [4] studies several kinds of risk factors affecting disease transmission, and Ref. [5] considers the evolutionary adaptability of the transmission objects in the process of transmission. Reference [6] uses the improved compartmental model to model the individual’s learning adaptive social behavior. A SEIADR model considering asymptomatic infection is proposed in Ref. [7], in which the global stability is analyzed using the qualitative theory of differential equation. Furthermore, three representative control effects are considered in Ref. [8], i.e. the feedback pulse terms that may be included in vaccination, treatment and infectious corpses. In Ref. [9], the age-structured SIR model with fixed incubation period is investigated theoretically. Reference [10] established compartmental models for two different groups of people including high-risk and low-risk infection, and investigated the optimal allocation measures under different vaccination-resource constraints. In addition, a substantial complete review of coupling behavior and disease modeling based on statistical physical methods is given in Ref. [15], [16].

With the emergence of a large number of asymptomatic infected cases, there have been some studies on asymptomatic infected population [7], [8], [14], [18], [21], there are three deficiencies in the existing research literature. First, the existing model considering asymptomatic infectors assumes that asymptomatic infectors are obtained from the exposed, while the existing evidence shows that asymptomatic infectors have not shown symptoms all the time. Unlike symptomatic infections, which have an incubation period, asymptomatic infections arise from susceptible populations rather than from exposed ones [22]. Second, some of the existing models addressed asymptomatic infection while not including the dead population explicitly [14], [18], [21], some other models did not incorporate the implementation effect of different intervention measures although considering the dead population [7], [8]. Third, the existing models do not take into account the differences in the final mortality rate caused by the adoption of different measures [7], [8], [14], [18], [21]. For the COVID-19, Ref. [23] assess the potential role of a number of public health measures – so-called non-pharmaceutical interventions (NPIs) – aimed at reducing contact rates in the population and thereby reducing transmission of the virus, i.e., mitigation and suppression measures.

Based on the characteristics of COVID-19 epidemic situation, this article improves the existing asymptomatic infector models according to these three problems. Firstly, in this model, the more realistic transmission path is considered, i.e., the asymptomatic infector population is generated from the susceptible population rather than from the exposed population. Secondly, the recovery group and the dead group are treated separately, and the dead group is regarded as an important indicator to reflect the effect of different response measures. Thirdly, compare the effectiveness of different prevention and response measures under different implementation time conditions, which may provide useful insights for selecting appropriate epidemic prevention and response measures in reality.

This article is organized as follows. In Section 2, a basic aSEI_1_I_2_RD epidemic model and it’s improvement bSEI_1_I_2_RD model are given. The main results of these models including preliminary theoretical analysis and numerical simulations are presented in Section 3. Section 4 discuss the implications of the results, Finally, concluding remarks of the paper are given in Section 5.

## II. Model Formulation

In what follows, infectors are divided into symptomatic infectors and asymptomatic infectors. The basic model without considering external disturbance factors and the improved model with considering external disturbance factors are presented. First, the final state of some population numbers is analyzed theoretically, and then the evolution of population numbers with different parameters is simulated numerically. We assume the initial total population number equals N, the maximal average contact number of one person in unit time is 10.

### A. Definitions and Notations

Definitions of related parameters and notations are listed in Table 1.

**TABLE 1:** Definitions of Related Parameters and Notations

Symbol	Quantity	Range
S(t)	number of susceptible individuals in t time	[0,N]
E(t)	number of exposed individuals in t time	[0,N]
I1(t)	number of symptomatic infected individuals in t time	[0,N]
I2(t)	number of asymptomatic infected individuals in t time	[0,N]
R(t)	number of resistive individuals in t time	[0,N]
D(t)	number of dead individuals in t time	[0,N]
$\alpha$	average number of susceptible persons contacted by infected persons in unit time	[0,10]
$\beta $	probability per unit time of transmitting the virus effectively between a carrier and a susceptible	[0,1]
$\sigma$	transition rate from the exposed to the symptomatic infectious, equals reciprocal of incubation period	[0,1]
$\gamma _{1} $	recovery rate from symptomatic infectious to resistive individuals	[0,1]
$\gamma _{2} $	recovery rate from asymptomatic infectious to resistive individuals	[0,1]
$\mu$	dead rate of symptomatic infectious	[0,1]
$\varepsilon$	interference coefficient used to disturb various quantity changes	[0,1]
Te	incubation period from infectious to symptomatic	[0,30]

### B. Assumptions

(1) All subpopulations number functions s (t), E (t), I1(t), I2(t), R (t), D (t) are continuously differentiable functions on interval $t\in [0,T]$;
(2) the number of exposed persons (infected but not ill) that a patient can infect in unit time is directly proportional to the total number of susceptible individuals S(t) in this environment. Proportion coefficient is the product of the number of people contacted in a unit time, the infection rate $\beta $, and the proportion of the infected people in the total population, i.e., the number of infected individuals per unit time is $(\alpha \beta (I_{1} +I_{2})/N)\ast S$;
(3) At time t, the number of people removed from symptomatic infectious in unit time is directly proportional to the number of symptomatic, and the proportion coefficient is $\gamma _{1} $;
(4) At time t, the number of people removed from asymptomatic infectious per unit time is directly proportional to the number of asymptomatic, and the proportion coefficient is $\gamma _{2} $;
(5) At time t, the number of deaths per unit time from symptomatic patients is directly proportional to the number of symptomatic, and the proportion coefficient is $\mu $;
(6) Basic reproductive number of infectious diseases is $R_{0} =\alpha \beta $.

### C. Basic Model and Improved Model

**FIGURE 1.**
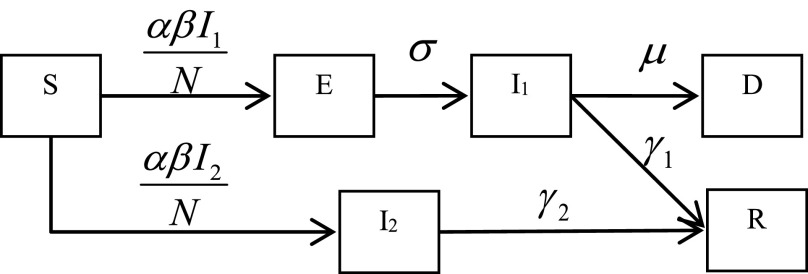
Schematic diagram of the SEI_1_I_2_RD model.

Considering the transmission path of SARS-CoV-2, first of all, from the susceptible subpopulation, after contact with the virus, it becomes a symptomatic infected individual during a period of incubation time, or become an asymptomatic infected person. The symptomatic infected person finally recovers after treatment, or the condition worsens to death, and the asymptomatic infected person finally recovers. Schematic diagram of the SEI_1_I_2_RD model can be described as Fig. 1.

The basic SEI_1_I_2_RD model can be formulated as follows:

\begin{align*} aSEI_{1}I_{2}RD:\begin{cases} \displaystyle \frac {dS}{dt}=-\frac {\alpha \beta (I_{1}+I_{2})}{N}S\\[0.4pc] \displaystyle \frac {dE}{dt}=\frac {\alpha \beta I_{1}}{N}S-\sigma E\\[0.4pc] \displaystyle \frac {dI_{1}}{dt}=\sigma E-(\gamma _{1}+\mu)I_{1}\\[0.4pc] \displaystyle \frac {dI_{2}}{dt}=\frac {\alpha \beta I_{2}}{N}S-\gamma _{2}I_{2}\\[0.4pc] \displaystyle \frac {dR}{dt}=\gamma _{1}~I_{1}+\gamma _{2}~I_{2}\\[0.4pc] \displaystyle \frac {dD}{dt}=\mu I_{1}\\ \displaystyle \end{cases}\end{align*}

It’s clear thar we get $\frac {dS}{dt}+\frac {dE}{dt}+\frac {dI_{1} }{dt}+\frac {dI_{2}}{dt}+\frac {dR}{dt}+\frac {dD}{dt}\textrm {=0}$ if we sum all the six equations together, which implies that in basic SEI_1_I_2_RD model, the total number of all six subpopulation is an constant, i.e., $S(t)+E(t)+I_{1} (t)+I_{2} (t)+R(t)+D(t)=N(t)\equiv N$.

Here the external disturb factors are not taken account into the basic SEI_1_I_2_RD model. If we add some external disturb factors into the into the basic SEI_1_I_2_RD model, we then get improved SEI_1_I_2_RD model as follows:

\begin{align*} bSEI_{1}I_{2}RD:\begin{cases} \displaystyle \frac {dS}{dt}=-\left [{\frac {\alpha \beta (I_{1}+I_{2})}{N}+\varepsilon }\right]S\\[0.5pc] \displaystyle \frac {dE}{dt}=\frac {\alpha \beta I_{1}}{N}S-(\sigma +\varepsilon)E\\[0.4pc] \displaystyle \frac {dI_{1}}{dt}=\sigma E-(\gamma _{1}+\mu +\varepsilon)I_{1}\\[0.4pc] \displaystyle \frac {dI_{2}}{dt}=\frac {\alpha \beta I_{2}}{N}S-(\gamma _{2}+\varepsilon)I_{2}\\[0.4pc] \displaystyle \frac {dR}{dt}=\gamma _{1}~I_{1}+\gamma _{2}~I_{2}-\varepsilon R\\[0.4pc] \displaystyle \frac {dD}{dt}=\mu I_{1}-\varepsilon D\\ \displaystyle \end{cases}\end{align*}

Note that for improved model, we can get $\frac {dS}{dt}+\frac {dE}{dt}+\frac {dI_{1}}{dt}+\frac {dI_{2} }{dt}+\frac {dR}{dt}+\frac {dD}{dt}= \varepsilon (S+E+I_{1} +I_{2} +R+D)$, it’s apparent that the right-hand value doesn’t equal constant. It implies that the total number of subpopulations is not an invariant anymore.

## III. Results

It’s extremely difficult to obtain analytical solution of aSEI_1_I_2_RD and bSEI_1_I_2_RD, the properties of the solution are analyzed firstly, and then the solution under different parameters is discussed by numerical simulation.

### A. Some Theorical Results of aSEI$_{{1}}~\text{I}_{{2}}$RD

(1) It can be seen from the first formula of aSEI_1_I_2_RD,
  $\frac {dS}{dt}=-\frac {\alpha \beta (I_{1} +I_{2})}{N}S < 0$, so $S(t)$ is a strictly monotonic decreasing function. At the same time, $0\le S(t)\le N$ is hold for all $t\ge 0$. Then there exists $S_{\infty } \in [0,N]$, $\lim \limits _{t\to \infty } S(t)=S_{\infty } $. That means the number of susceptible will eventually become stable.
(2) Add the 1st, 2nd and 4th equations of aSEI_1_I_2_RD model, we can get $\frac {d(S+E+I_{2})}{dt}=-\sigma E-\gamma _{2} I_{2} $, Integral on both sides leads to $\int _{0}^{T} {\frac {d(S+E+I_{2})}{dt}dt=-\sigma \int _{0}^{T} E dt-\gamma _{2} \int _{0}^{T} {I_{2} (t)dt}} $, Left hand equals $S(T)+E(T)+I_{2} (t)-S(0)-E(0)-I_{2} (0)$, whose upper bound is $6N$. Let $T\to \infty $, we get $\int _{0}^\infty {E(t)} dt+\int _{0}^\infty {I_{2} (t)} \le \frac {6N}{\min \{\sigma,\gamma _{2} \}}$. Because $E(t)\ge 0$ and $I_{2} (t)\ge 0,E(t)$ and $I_{2} (t)$ are continuously differentiable, we can get $\lim \limits _{t\to \infty } E(t)=0$, $\lim \limits _{t\to \infty } I_{2} (t)=0$. This means that all exposed people will recover or die, and all asymptomatic infected people will recover.
(3) Add the first four formulas of aSEI_1_I_2_RD model, we can get $\frac {d(S+E+I_{1} +I_{2})}{dt}=-(\gamma _{1} +\mu)I_{1} -\gamma _{2} I_{2} $. Integral on both sides leads to $\int _{0}^{T} \frac {d(S+E+I_{1} +I_{2})}{dt}dt=-(\gamma _{1} +\mu)\,\,\int _{0}^{T} {I_{1} (t)} dt-\gamma _{2} \int _{0}^{T} {I_{2} (t)dt}$. Left side equals $S(T)+E(T)+I_{1} (t)+I_{2} (t)-S(0)-E(0)-I_{1} (0)-I_{2} (0)$. Upper bound is $8N$. Let $T\to \infty $, we get $\int _{0}^\infty {I_{1} (t)} dt+\int _{0}^\infty {I_{2} (t)} \le \frac {8N}{\min \{\gamma _{1} +\mu,\gamma _{2} \}}$, Because $I_{1} (t)\ge 0,I_{1} (t)$ are continuously differentiable, we can get $\lim \limits _{t\to \infty } I_{1} (t)=0$. This means that all those symptomatic infectious will recover or die eventually.
(4) Because $S(t)$ is a strictly monotone decreasing function, it can be seen from the second formula of aSEI_1_I_2_RD model, when $\frac {\alpha \beta I_{1}}{N}S-\sigma E=0$, i.e., $S=\frac {\sigma EN}{\alpha \beta I_{1}}$, the number of exposed individuals $E(t)$ will reach it’s maximum value.

### B. Numerical Simulation Results of aSEI$_{{1}}~\text{I}_{{2}}$ RD

In the part of numerical simulation, we consider three different response measures: m1. mitigation measures, m2. suppression measures, and m3. medical promotion. Mitigation measures and suppression measures are reflected by the average number of contacts $\alpha $, medical promotion is reflected by the recovery rate $\gamma _{1} $ and $\gamma _{2} $. Nevertheless, according to the existing experience, asymptomatic infections may be self-healing or very easy to cure. Therefore, in simulation analysis, we assume that the recovery rate of asymptomatic infection $\gamma _{2} $ is constant, and the effect of medical promotion is mainly reflected through the recovery rate of symptomatic infection $\gamma _{1} $. We will let $\gamma _{1} $ change from a small value (0.05) to a large value (0.8), which indicates the progress of medical level. For each measure, the evolution trend of epidemic under the initial population distribution structure of four different groups are discussed. In the numerical simulation, different initial population distribution conditions are corresponding to different development periods of the epidemic.

For example, the population distribution of group 1 is $N=10000,S(0)=9999$, $E(0)=1$, $I_{1} (0)=0$, $I_{2} (0)=0$, $R(0)=0$, $D(0)=0$, corresponding to the initial stage of epidemic development. The population distribution of group 2 is $N=10000,S(0)=9000$, $E(0)=1000$, $I_{1} (0)=0$, $I_{2} (0)=0$, $R(0)=0$, $D(0)=0$, corresponding to the early stage of epidemic development. The population distribution of group 3 is $N=10000,S(0)=9000$, $E(0)=1000$, $I_{1} (0)=0$, $I_{2} (0)=0$, $R(0)=0$, $D(0)=0$, corresponding to the middle stage of epidemic development, and the population distribution of group 4 is $N=10000,S(0)=8500$, $E(0)=0$, $I_{1} (0)=1000$, $I_{2} (0)=500$, $R(0)=0$, $D(0)=0$, corresponding to the middle and later stage of epidemic development. The incubation period Te is taken as 14(days), i.e., the transition rate $\sigma =\textrm {1/14}$ in the following simulation according to the COVID-19 reports [23]. The values of other parameters, i.e., $\alpha,\beta,\gamma _{1},\gamma _{2} $ and $\mu $, are verified according to related literature ([21] ~ [23]).

The parameter settings of the numerical simulation and the corresponding results are shown in Table 2.

**FIGURE 2.**
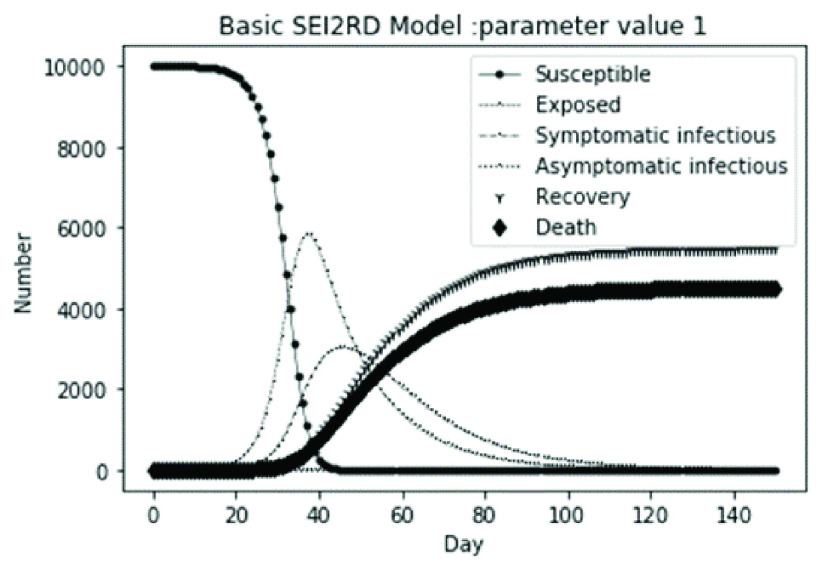
Simulation of aSEI_1_I_2_RD model on parameter value 1.

**FIGURE 3.**
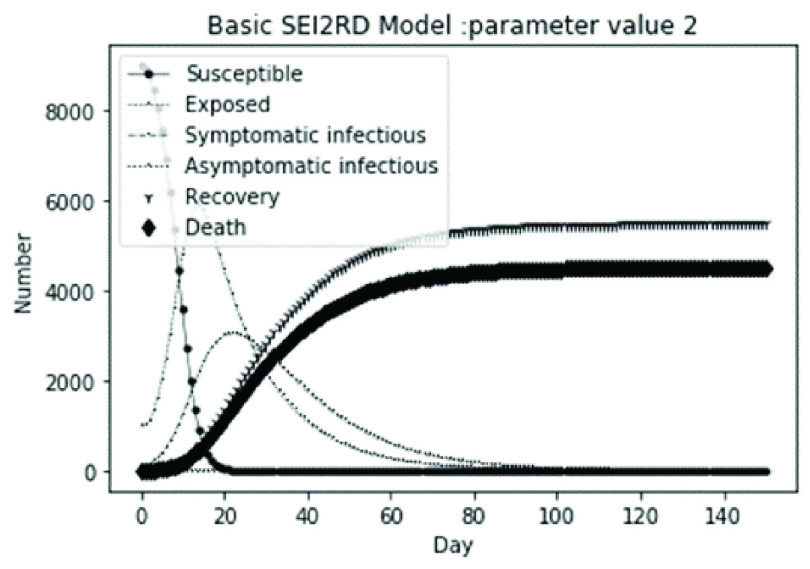
Simulation of aSEI_1_I_2_RD model on parameter value 2.

**FIGURE 4.**
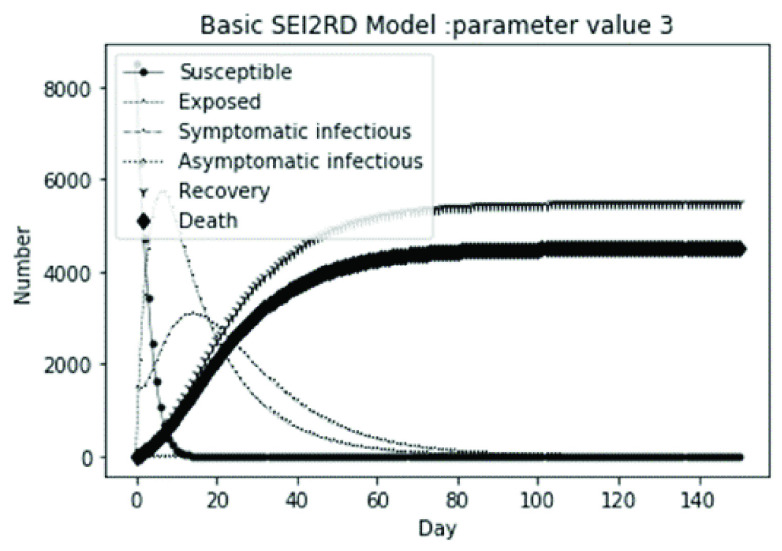
Simulation of aSEI_1_I_2_RD model on parameter value 3.

**FIGURE 5.**
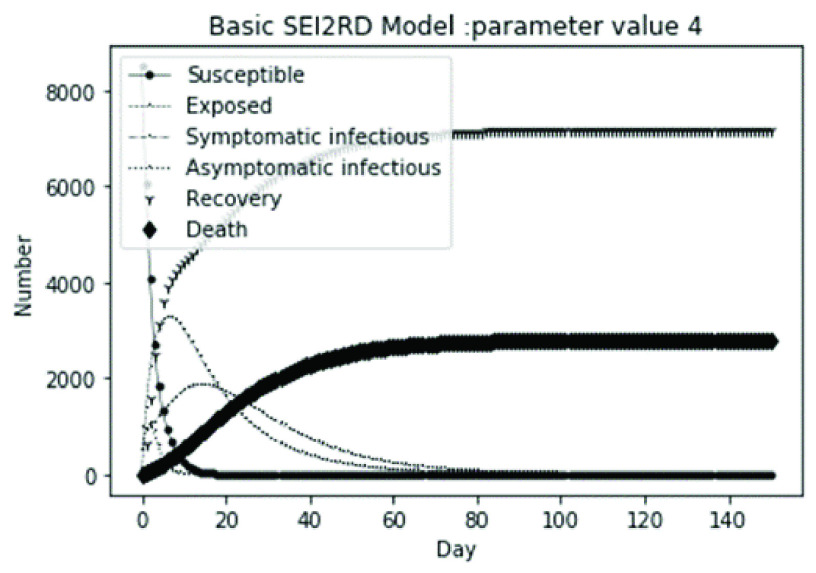
Simulation of aSEI_1_I_2_RD model on parameter value 4.

**FIGURE 6.**
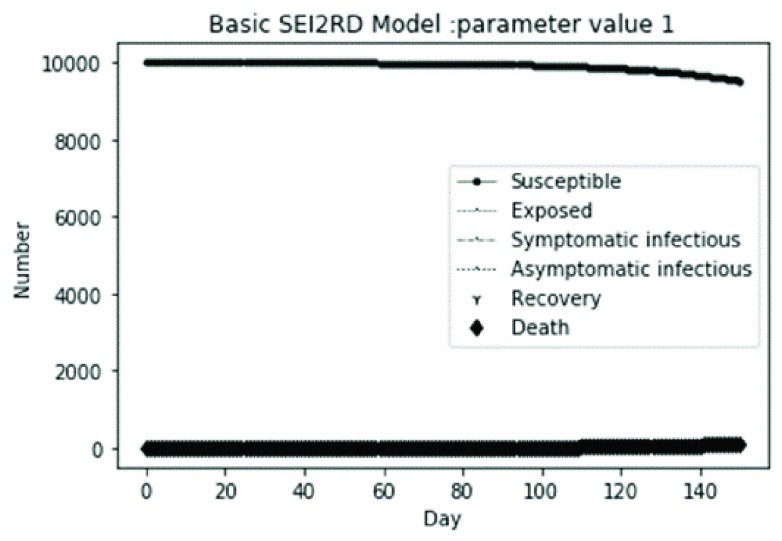
Simulation of aSEI_1_I_2_RD model on parameter value 5.

**FIGURE 7.**
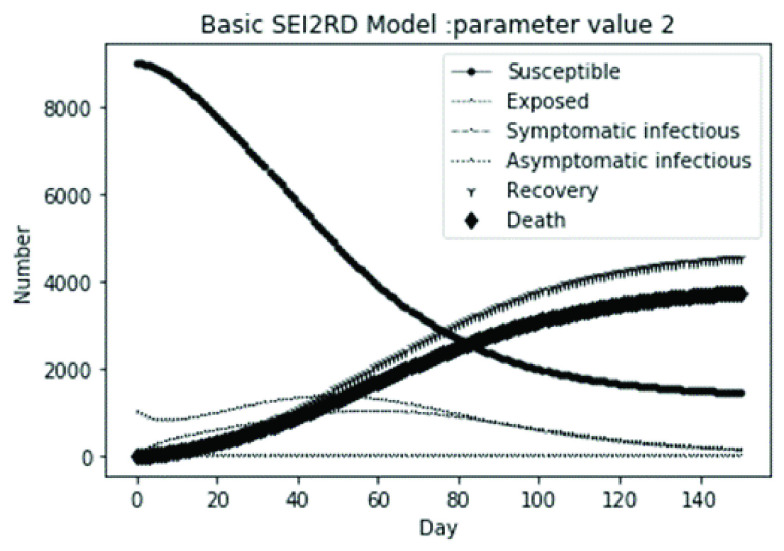
Simulation of aSEI_1_I_2_RD model on parameter value 6.

**FIGURE 8.**
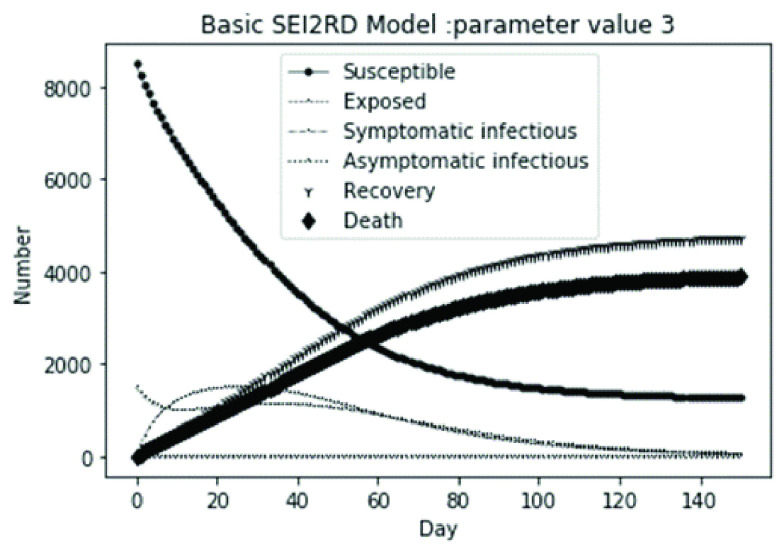
Simulation of aSEI_1_I_2_RD model on parameter value 7.

**FIGURE 9.**
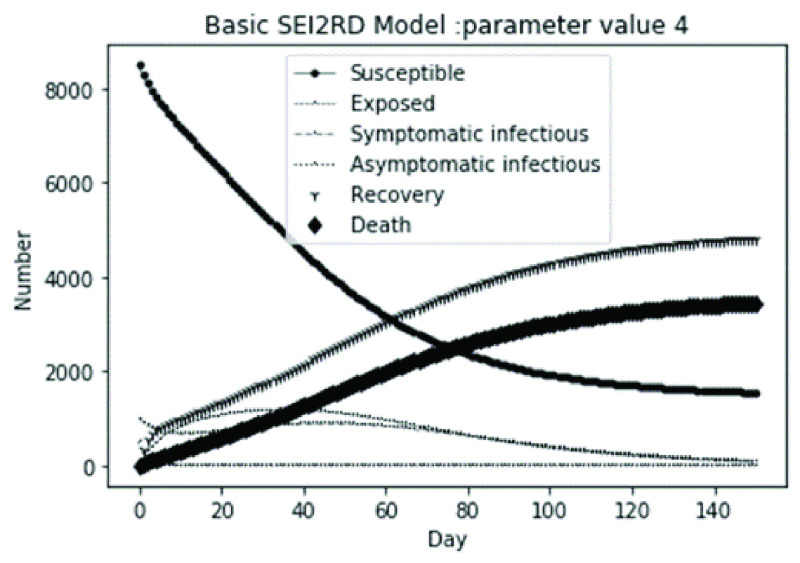
Simulation of aSEI_1_I_2_RD model on parameter value 8.

**FIGURE 10.**
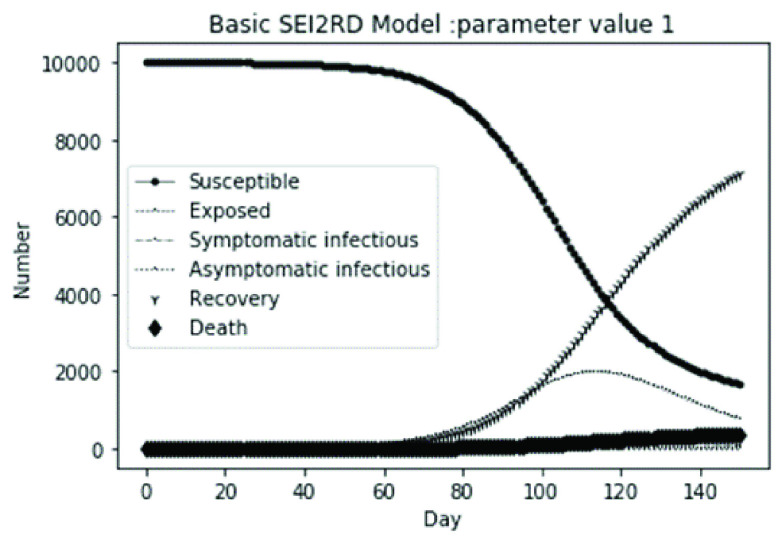
Simulation of aSEI_1_I_2_RD model on parameter value 9.

**FIGURE 11.**
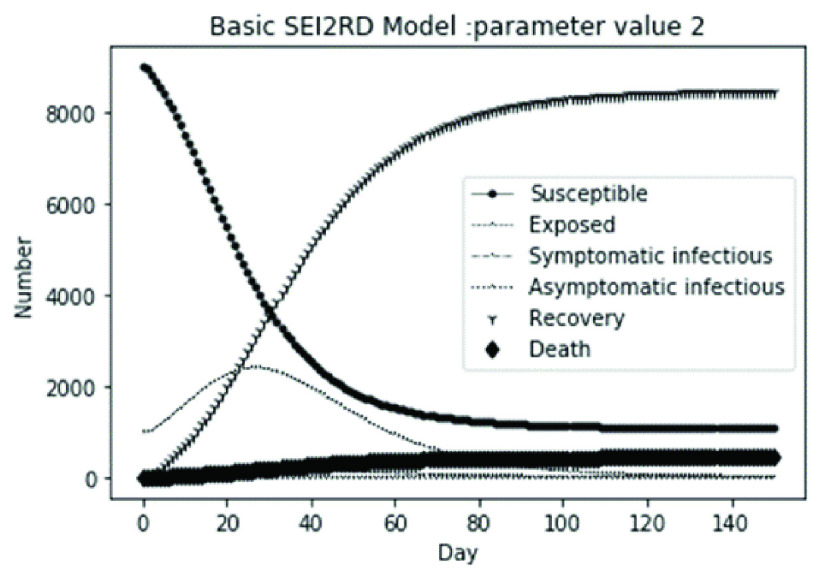
Simulation of aSEI_1_I_2_RD model on parameter value 10.

**FIGURE 12.**
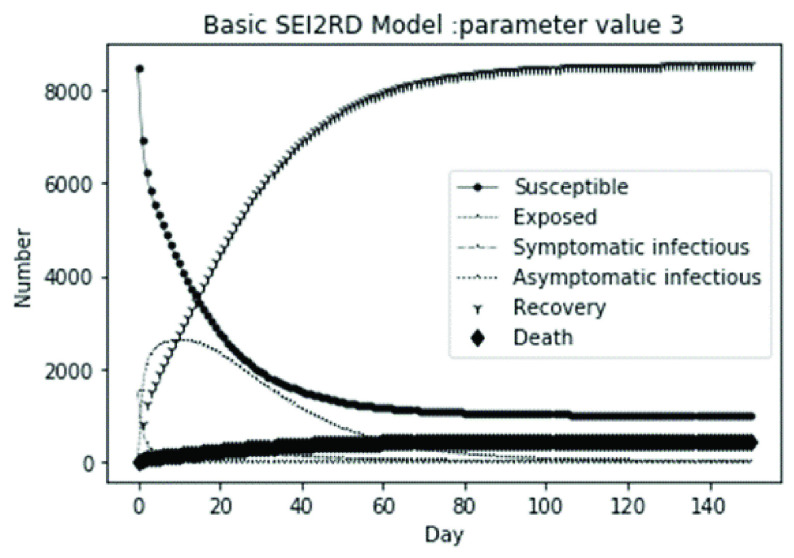
Simulation of aSEI_1_I_2_RD model on parameter value 11.

**FIGURE 13.**
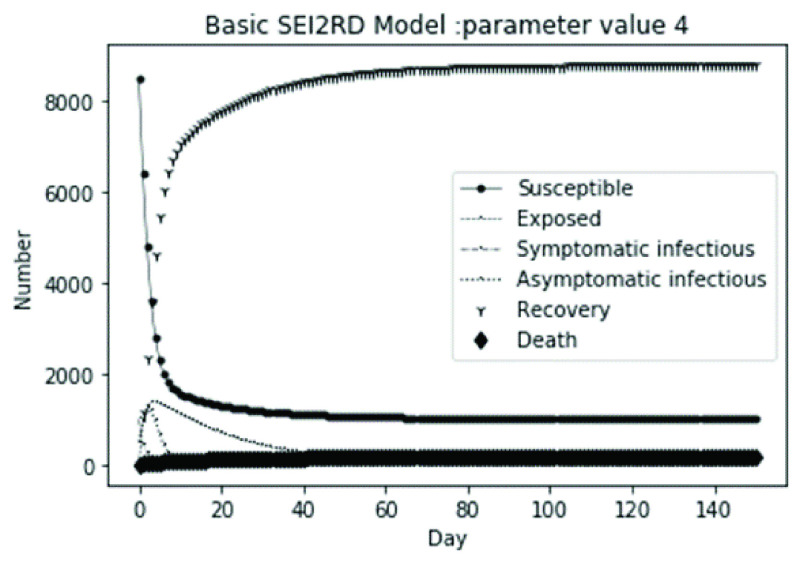
Simulation of aSEI_1_I_2_RD model on parameter value 12.

(1) The values of the first group of parameters are as follows: $\alpha =10$, $\beta =0.2$, $\sigma =\textrm {1/14}$, $\gamma _{1} =0.05$, $\gamma _{2} =0.85$, $\mu =0.041$; $N=10000$. $S(0)=9999$, $E(0)=1$, $I_{1} (0)=0$, $I_{2} (0)=0$, $R(0)=0$, $D(0)=0$. Final values are S(T) = 0, E(T) = 2, I1(T) = 7, I2(T) = 0, R(T) = 5489, D(T) = 4501. The numerical simulation results are shown in Fig. 2.
(2) The values of the second group of parameters are as follows: $\alpha =10$, $\beta =0.2$, $\sigma =\textrm {1/14}$, $\gamma _{1} =0.05$, $\gamma _{2} =0.85$, $\mu =0.041$; $N=10000$. $S(0)=9000$, $E(0)=1000$, $I_{1} (0)=0$, $I_{2} (0)=0$, $R(0)=0$, $D(0)=0$. Final values are S(T) = 0, E(T) = 0, I1(T) = 1, I2(T) = 0, R(T) = 5493, D(T) = 4505. The numerical simulation results are shown in Fig. 3.
(3) The values of the third group of parameters are as follows: $\alpha =10$, $\beta =0.2$, $\sigma =\textrm {1/14}$, $\gamma _{1} =0.05$, $\gamma _{2} =0.85$, $\mu =0.041$; $N=10000$. $S(0)=8500$, $E(0)=0$, $I_{1} (0)=1500$, $I_{2} (0)=0$, $R(0)=0$, $D(0)=0$. Final values are S(T) = 0, E(T) = 0, I1(T) = 1, I2(T) = 0, R(T) = 5493, D(T) = 4505. The numerical simulation results are shown in Fig. 4.
(4) The values of the fourth group of parameters are as follows: $\alpha =10$, $\beta =0.2$, $\sigma =\textrm {1/14}$, $\gamma _{1} =0.05$, $\gamma _{2} =0.85$, $\mu =0.041$; $N=10000$. $S(0)=8500$, $E(0)=0$, $I_{1} (0)=1000$, $I_{2} (0)=500$, $R(0)=0$, $D(0)=0$. Final values are S(T) = 0, E(T) = 0, I1(T) = 1, I2(T) = 0, R(T) = 7199, D(T) = 2800. The numerical simulation results are shown in Fig. 5.
(5) Considering the comparison of different response measures, the values of the fifth group of parameters are as follows: assuming that strict control measures are taken to socially isolate residents, the average number of contacts reduces from $\alpha =10$ to $\alpha =1$, and other parameters are the same as those of the first group. Final values are S(T) = 9518, E(T) = 165, I1(T) = 93, I2(T) = 0, R(T) = 123, D(T) = 101. The numerical simulation results are shown in Fig. 6.
(6) Considering the comparison of different response measures, the values of the sixth group of parameters are as follows: assuming that strict control measures are taken to socially isolate residents, the average number of contacts reduces from $\alpha =10$ to $\alpha =1$, and other parameters are the same as those of the first group. Final values are S(T) = 1448, E(T) = 106, I1(T) = 132, I2(T) = 0, R(T) = 4568, D(T) = 3746. The numerical simulation results are shown in Fig. 7.
(7) Considering the comparison of different response measures, the values of the 7th group of parameters are as follows: assuming that strict control measures are taken to socially isolate residents, the average number of contacts reduces from $\alpha =10$ to $\alpha =1$, and other parameters are the same as those of the first group. Final values are S(T) = 1275, E(T) = 41, I1(T) = 54, I2(T) = 0, R(T) = 4742, D(T) = 3888. The numerical simulation results are shown in Fig. 8.
(8) Considering the comparison of different response measures, the values of the 8th group of parameters are as follows: assuming that strict control measures are taken to socially isolate residents, the average number of contacts reduces from $\alpha =10$ to $\alpha =1$, and other parameters are the same as those of the first group. Final values are S(T) = 1556, E(T) = 74, I1(T) = 90, I2(T) = 0, R(T) = 4828, D(T) = 3451. The numerical simulation results are shown in Fig. 9.
(9) Assuming the level of recovery rate $\gamma _{1} $ increases from 0.05 to 0.8 due to the improvement of treatment level, $\gamma _{2} $ remains unchanged, other parameters are the same as those of the first group. Final values are S(T) = 1684, E(T) = 758, I1(T) = 68, I2(T) = 0, R(T) = 7165, D(T) = 365. The numerical simulation results are shown in Fig. 10.
(10) Assuming the level of recovery rate $\gamma _{1} $ increases from 0.05 to 0.8 due to the improvement of treatment level, $\gamma _{2} $ remains unchanged, other parameters are the same as those of the first group. Final values are S(T) = 1082, E(T) = 10, I1(T) = 1, I2(T) = 0, R(T) = 8473, D(T) = 434. The numerical simulation results are shown in Fig. 11.
(11) Assuming the level of recovery rate $\gamma _{1} $ increases from 0.05 to 0.8 due to the improvement of treatment level, $\gamma _{2} $ remains unchanged, other parameters are the same as those of the first group. Final values are S(T) = 1001, E(T) = 4, I1(T) = 0, I2(T) = 0, R(T) = 8556, D(T) = 439. The numerical simulation results are shown in Fig. 12.
(12) Assuming the level of recovery rate $\gamma _{1} $ increases from 0.05 to 0.8 due to the improvement of treatment level, $\gamma _{2} $ remains unchanged, other parameters are the same as those of the first group. Final values are S(T) = 1018, E(T) = 1, I1(T) = 0, I2(T) = 0, R(T) = 8810, D(T) = 171. The numerical simulation results are shown in Fig. 13.

**TABLE 2:** Correspondence Between Numerical Simulation Parameters and Results

	Basic model: aSEI_1_I_2_RD			Improved model: bSEI_1_I_2_RD
response measures				
population distribution	m1	m2	m3	m3
Group 1	Fig. 2.	Fig. 6.	Fig. 10.	Fig. 14.
Group 2	Fig. 3.	Fig. 7.	Fig. 11.	Fig. 15.
Group 3	Fig. 4.	Fig. 8.	Fig. 12.	Fig. 16.
Group 4	Fig. 5.	Fig. 9.	Fig. 13.	Fig. 17.

### C. Numerical Simulation Results of bSEI$_{{1}}~\text{I}_{{2}}$ RD

Similar to aSEI_1_I_2_RD model, related results $E(t)\to 0$, $I_{1} (t)\to 0$, $I_{2} (t)\to 0$, can be obtained from the theoretical analysis of bSEI_1_I_2_RD model. Here we directly carry out numerical simulation analysis. Only the parameters of the first group to the fourth group of population initial distribution structure corresponding to the mitigation measures (m1) are selected for simulation.

**FIGURE 14.**
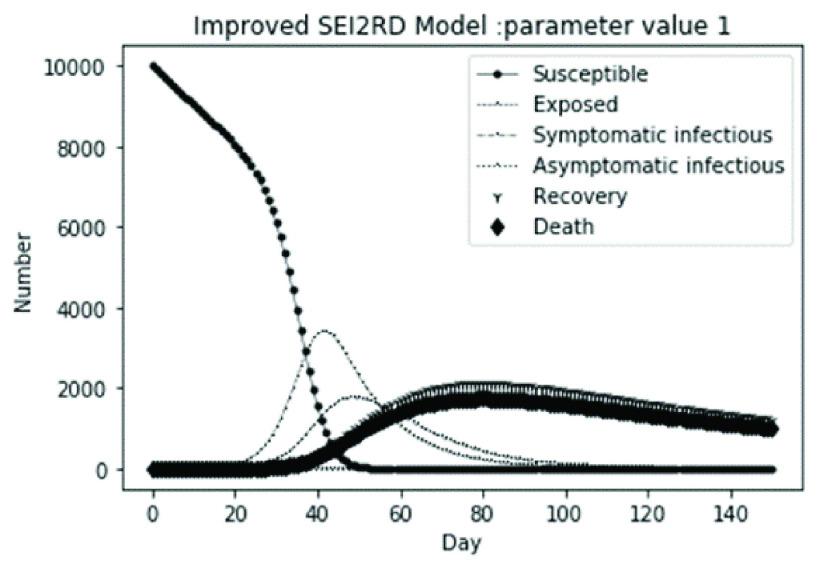
Simulation of bSEI_1_I_2_RD model on parameter value 1.

**FIGURE 15.**
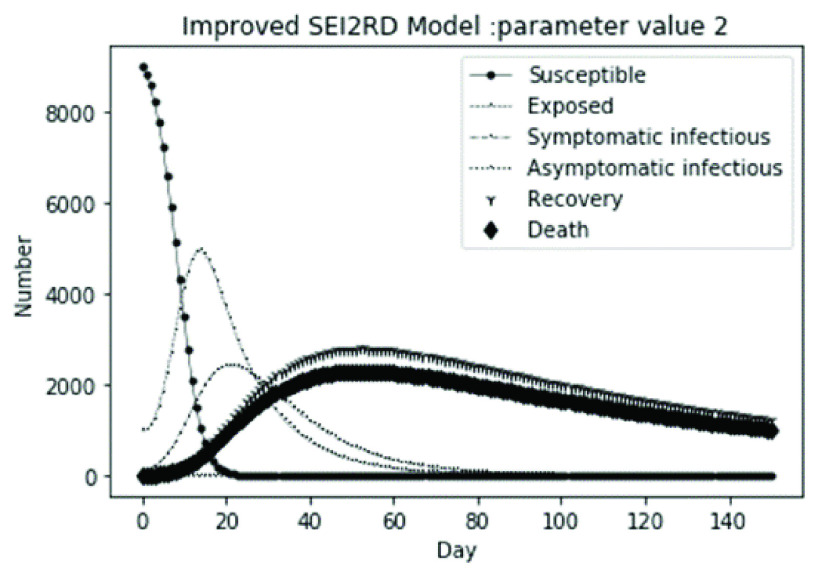
Simulation of bSEI_1_I_2_RD model on parameter value 2.

**FIGURE 16.**
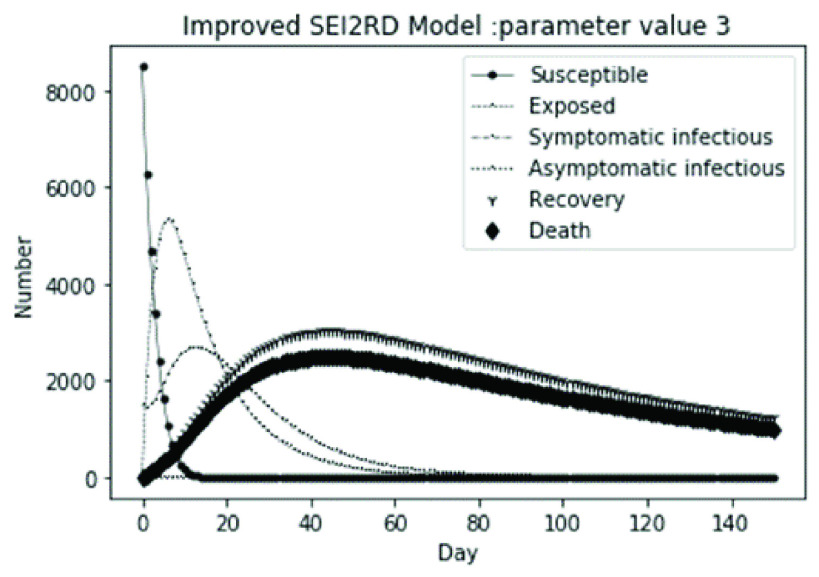
Simulation of bSEI_1_I_2_RD model on parameter value 3.

**FIGURE 17.**
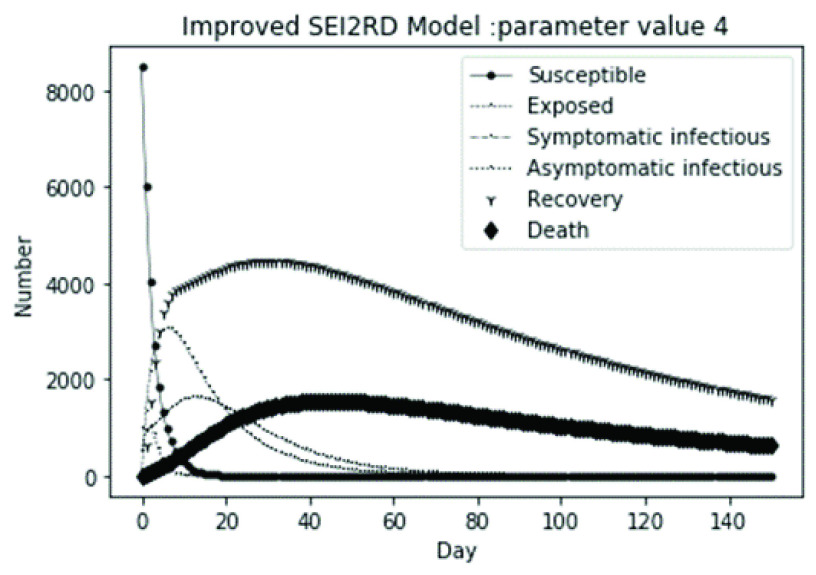
Simulation of bSEI_1_I_2_RD model on parameter value 4.

(1) The values of the first group of parameters are as follows: $\alpha =10$, $\beta =0.2$, $\sigma =\textrm {1/14}$, $\gamma _{1} =0.05$, $\gamma _{2} =0.85$, $\mu =0.041$;$N=10000$. $S(0)=9999$, $E(0)=1$, $I_{1} (0)=0$, $I_{2} (0)=0$, $R(0)=0$, $D(0)=0$; Interference coefficient $\varepsilon =0.01$. Final values are S(T) = 0, E(T) = 1, I1(T) = 2, I2(T) = 0, R(T) = 1224, D(T) = 1004. The numerical simulation results are shown in Fig. 14.
(2) The values of the second group of parameters are as follows: $\alpha =10$, $\beta =0.2$, $\sigma =\textrm {1/14}$, $\gamma _{1} =0.05$, $\gamma _{2} =0.85$, $\mu =0.041$;$N=10000$. $S(0)=9000$, $E(0)=1000$, $I_{1} (0)=0$, $I_{2} (0)=0$, $R(0)=0$, $D(0)=0$; Interference coefficient $\varepsilon =0.01$. Final values are S(T) = 0, E(T) = 0, I1(T) = 0, I2(T) = 0, R(T) = 1226, D(T) = 1005. The numerical simulation results are shown in Fig. 15.
(3) The values of the third group of parameters are as follows: $\alpha =10$, $\beta =0.2$, $\sigma =\textrm {1/14}$, $\gamma _{1} =0.05$, $\gamma _{2} =0.85$, $\mu =0.041$; $N=10000$. $S(0)=8500$, $E(0)=0$, $I_{1} (0)=1500$, $I_{2} (0)=0$, $R(0)=0$, $D(0)=0$; Interference coefficient $\varepsilon =0.01$. Final values are S(T) = 0, E(T) = 0, I1(T) = 0, I2(T) = 0, R(T) = 1226, D(T) = 1005. The numerical simulation results are shown in Fig. 16.
(4) The values of the fourth group of parameters are as follows: $\alpha =10$, $\beta =0.2$, $\sigma =\textrm {1/14}$, $\gamma _{1} =0.05$, $\gamma _{2} =0.85$, $\mu =0.041$; $N=10000$. $S(0)=8500$, $E(0)=0$, $I_{1} (0)=1000$, $I_{2} (0)=500$, $R(0)=0$, $D(0)=0$; Interference coefficient $\varepsilon =0.01$. Final values are S(T) = 0, E(T) = 0, I1(T) = 0, I2(T) = 0, R(T) = 1601, D(T) = 630. The numerical simulation results are shown in Fig. 17.

## IV. Discussion

In this article, a dynamic model is presented to describe the characteristics of relative long incubation period and a large number of asymptomatic infections such as COVID-19 epidemic. we investigate the effect at three dimensions, i.e., different initial population distribution structure, response measures, and external disturbance on the evolutionary output of the subpopulations. The initial population distribution structures are divided as group1, group2, group 3 and group 4 listed in Table 1. We take account for three representative response measures such as: m1. mitigation measures, m2. suppression measures, and m3. medical promotion. In our numerical analysis, all these dimensions are embodied by different parameter settings. Different parameter combinations listed in Table 1. will produce different system outputs showed from Fig. 2. to Fig. 17. We’ll discuss our findings from four aspects.

### A. Evolution Trend of Epidemic Situation Under Different Initial Population Distribution Structure

From Fig. 2. to Fig. 5., with the change of initial population distribution structure, the final value of each subpopulation of the system changes little, which affects the time of evolution to stable state. For example, in Fig. 2., Fig 3. and Fig. 4., the time of symptomatic infection changing to stable state are about 100, 90, and 80 days respectively, which mean that Fig. 2. is the slowest, Fig. 3. is the second, and Fig. 4. is the fastest. This is because in Fig. 2., susceptible subpopulation $S(0)=9999$, exposed subpopulation $E(0)=1$, there is only one exposed person at the beginning. In Fig. 3., the susceptible subpopulation $S(0)=9000$, exposed subpopulation $E(0)=1000$. In Fig. 4., the susceptible subpopulation $S(0)=8500$, the symptomatic infected subpopulation $I_{1} (0)=1500$. Fig. 4. is equivalent to the intermediate state in Fig. 2., so the time converging to stable state is the shortest. However, Fig. 5. is quite different from the previous three figures. The final death toll of Fig. 5. is about 62% of the previous Fig. 2. to Fig. 4. (2800 / 4500 ≈ 62.153%). The results showed that under fixed prevention and control measures and medical conditions, the number of initial exposure $E(0)$ and the number of symptomatic infectious $I_{1} (0)$ had little effect on the mortality, while the number of asymptomatic infectious $I_{2} (0)$ had a significant effect on the final mortality. These scenarios are determined by the parameters $\alpha $, $\beta $, $\gamma _{1} $ and $\gamma _{2} $ demonstrated in Fig 2 to Fig 5. Because the recovery rate of asymptomatic infectious is high ($\gamma _{2} =0.85$), the number of asymptomatic infections will affect the final death toll significantly and positively.

### B. Impact of Mitigation and Suppression Measures

The average number of contacts in Fig. 2.–Fig. 5. is $\alpha =10$ and changes to $\alpha =1$ in Fig. 6.–Fig. 9. $\alpha =10$ in Fig. 2.–Fig. 5. indicates that the average number of susceptible people contacted by the infected is 10, which corresponds to the relatively loose prevention and control measures in reality. For example, European and American countries do not limit social distance at the beginning of the epidemic, and there is no strict isolation and blockade, which is equivalent to mitigation measures. If we reduce this index to $\alpha =1$, corresponding to the more strict prevention and control measures in reality, for example, the closure measures taken by China to strictly restrict residents from going out after the outbreak of the epidemic, which is equivalent to the suppression measures, as shown in Fig. 6.–Fig. 9. The results show that the initial structure of the population has a significant impact on the final effect under the condition of suppression measures.

Fig. 6. shows that there is only one exposed person at the beginning ($E(0)=1)$, and the final death toll is the lowest (D (T) = 101). Fig. 7. to Fig. 9. correspond to the situation of more initial exposed persons ($E(0)=1000)$, symptomatic infectious ($I_{1} (0)=1500)$, and asymptomatic infectious ($I_{2} (0)=500)$, respectively, the final death toll is significantly higher than that in the first case (D(T) is 3746, 3888, and 3451 respectively). It shows that in the early stage of the epidemic, if strict isolation and blockade measures are implemented, the death toll can be effectively reduced, but in the later stage, the effect is poor. Comparatively speaking, the implementation of loose mitigation measures, no matter in the early stage and the middle stage of the epidemic, has little difference in effect (The death toll is 4501, 4505, and 4505 in Fig. 2.–Fig. 4.).

It is worth noting that the final death toll in Fig. 5. is 2800, which is significantly less than that in Fig. 9. (3451). It shows that even though more strict measures of suppression are adopted, the effect in the later stage of the epidemic development is not as effective as the loose measures. This proves from one side that “group immunity” can play a more obvious role in epidemic control, but “group immunity” may need to pay a considerable cost in the early stage of the epidemic, for example, a higher number of deaths. It is not easy for countries with serious epidemic situation to choose between mitigation measures and suppression measures.

### C. Impact of Medical Treatment Level

The level of medical treatment is reflected by recovery rate $\gamma _{1} $ and $\gamma _{2} $ corresponding to Fig. 10. to Fig. 13. We have improved the recovery rate $\gamma _{1} $ from 0.05 to 0.8, recovery rate $\gamma _{2} $ stands the same level, and observe the output results as shown in Fig. 10–Fig. 13. When the medical treatment level is improved, the final death toll will be significantly reduced. In the four cases of susceptible, initial exposure, symptomatic infection and asymptomatic infection are (9999, 1, 0, 0), (9000, 1000, 0, 0), (8500, 0,1500, 0), (8500, 0,1000, 500), the final death toll will be 0, 62, 91, 61, respectively.

This shows that even if the proportion of initial exposure or initial infection is relatively high, the death caused by the final epidemic can be well controlled under the effective medical level, which is consistent with our experience and cognition. At the same time, it also shows that efforts to improve the level of medical treatment, including the development of more effective drugs and vaccines, will play a crucial role in effectively reducing mortality.

### D. The Influence of Model Structure on Subpopulations’ Change

According to Fig. 14. to Fig. 17., which are conducted from improved model bSEI_1_I_2_RD, the total population is no longer kept at a fixed value due to the consideration of external disturbance factors of each type of subpopulation, and the final number of recovery and death is significantly reduced compared with Fig. 2. to Fig. 5. However, the trend of population change of each type is consistent with the situation without considering disturbance factors, which shows that the structure of the model plays a decisive role on the population quantitative trends.

## V. Conclusion

COVID-19 epidemic has a relative long incubation period and a large number of asymptomatic infections, which are significant features of SARS-CoV-2. In view of the lack of consideration on these distinctive characteristics of COVID-19 caused by SARS-CoV-2 in the existing literature, the epidemic of COVID-19 is modeled based on the classical compartmental model. The evolution trend of the number of subpopulations are analyzed theoretically, and the potential effects of three different epidemic response measures, namely mitigation measures, suppression measures and medical treatment promotion are discussed.

The results of numerical simulation show that the effect of mitigation measures and suppression measures depends on the timing of the implementation of measures. The effect of adopting suppression measures in initial stages on effectively reducing the number of deaths is significantly better than that in the middle and later stages, while the effect of the implementation timing of mitigation measures is not so obvious. At the same time, the improvement of medical treatment has a positive effect on reducing the number of deaths, no matter when it is used.

The results suggest that the use of mitigation measures and suppression measures should be combined with the specific situation of local epidemic development. In the future, we can further investigate the existence and stability of the model solution in theory, calibrate the parameters of the model and expand the validity of the model by using the actual data. In addition, we can consider the feedback incentive effect of different measures and the delay effect of immune measures.
